# Artificial Grammar Learning Capabilities in an Abstract Visual Task Match Requirements for Linguistic Syntax

**DOI:** 10.3389/fpsyg.2018.01210

**Published:** 2018-07-24

**Authors:** Gesche Westphal-Fitch, Beatrice Giustolisi, Carlo Cecchetto, Jordan S. Martin, W. Tecumseh Fitch

**Affiliations:** ^1^Department of Cognitive Biology, University of Vienna, Vienna, Austria; ^2^Department of Neurology, Medical University of Vienna, Vienna, Austria; ^3^Department of Psychology, University of Milan-Bicocca, Milan, Italy; ^4^Structures Formelles du Langage (Unité Mixte de Recherche CNRS and Université Paris 8), Paris, France; ^5^Department of Anthropology, Emory University, Atlanta, GA, United States

**Keywords:** artificial grammar learning, working memory, formal language theory, long-distance dependencies, mildly context sensitive grammars

## Abstract

Whether pattern-parsing mechanisms are specific to language or apply across multiple cognitive domains remains unresolved. Formal language theory provides a mathematical framework for classifying pattern-generating rule sets (or “grammars”) according to complexity. This framework applies to patterns at any level of complexity, stretching from simple sequences, to highly complex tree-like or net-like structures, to any Turing-computable set of strings. Here, we explored human pattern-processing capabilities in the visual domain by generating abstract visual sequences made up of abstract tiles differing in form and color. We constructed different sets of sequences, using artificial “grammars” (rule sets) at three key complexity levels. Because human linguistic syntax is classed as “mildly context-sensitive,” we specifically included a visual grammar at this complexity level. Acquisition of these three grammars was tested in an artificial grammar-learning paradigm: after exposure to a set of well-formed strings, participants were asked to discriminate novel grammatical patterns from non-grammatical patterns. Participants successfully acquired all three grammars after only minutes of exposure, correctly generalizing to novel stimuli and to novel stimulus lengths. A Bayesian analysis excluded multiple alternative hypotheses and shows that the success in rule acquisition applies both at the group level and for most participants analyzed individually. These experimental results demonstrate rapid pattern learning for abstract visual patterns, extending to the mildly context-sensitive level characterizing language. We suggest that a formal equivalence of processing at the mildly context sensitive level in the visual and linguistic domains implies that cognitive mechanisms with the computational power to process linguistic syntax are not specific to the domain of language, but extend to abstract visual patterns with no meaning.

## Introduction

Recent years have seen the rise of a new approach to investigating higher cognition in humans and other animals, specifically the ability to recognize patterns of various types and complexity (Saffran et al., 1996; Marcus et al., 1999; Fitch and Friederici, 2012; ten Cate and Okanoya, 2012).

These studies have examined patterns at different levels of complexity (Fitch and Hauser, 2004; Uddén et al., 2012), across different sensory and cognitive domains including spoken, musical or visual stimuli (Saffran et al., 1999, 2007), across different categories of humans [e.g., infants, normal adults, or patients (Reber and Squire, 1999; Saffran et al., 1999)], and across different species of birds and mammals (e.g., Gentner et al., 2006; Murphy et al., 2008; Stobbe et al., 2012; Wilson et al., 2013; Sonnweber et al., 2015). Most of these studies use some variant of “artificial grammar learning” (AGL: Reber, 1967), in which some specific rule, chosen by the experimenter, is used to generate stimuli which are then presented to participants during an exposure stage. Then, during a test stage, novel stimuli that either follow the chosen pattern or violate it in some way are used to probe what (if anything) the participant learned about the stimuli and the underlying pattern.

Because different pattern-generating rule systems (technically termed “grammars”) can be objectively ranked using the mathematical framework of formal language theory (Jäger and Rogers, 2012), patterns of success or failure can be used to evaluate the abilities of different species or human populations to recognize and generalize rules at different levels of complexity (Fitch and Friederici, 2012; Wilson et al., 2013) along with the brain circuitry used to process different types of patterns (Friederici et al., 2006; Pulvermüller, 2010). Similarities in human pattern processing across different sensory domains suggest that pattern-processing abilities generalize across domains and modalities and are not specific to language (Saffran et al., 1999, 2007). Furthermore, comparative analyses have led to the proposal that (adult) humans possess the ability to process rule systems at a higher formal level of complexity than do animals (Fitch and Hauser, 2004; Fitch, 2014). However, the degree to which pattern perception is domain-specific or modality-general remains debated (Frost et al., 2015), as does the degree to which failures of non-human species to master certain rule types truly reflects cognitive limitations at the rule-learning level (ten Cate and Okanoya, 2012).

There are several core empirical issues that hinder resolution of these multiple open debates. The first concerns whether differences found between species truly represent differences in rule appreciation, or could result from simpler differences such as working memory limitations (a core issue for any type of complex auditory stimulus). The second concerns the type of items used to generate stimuli, which vary considerably across studies. For acoustic stimuli, human syllables may be more salient or discriminable to people than to animals; similarly bird or monkey calls elements may be more meaningful or arousing to conspecifics (Chen et al., 2015). Furthermore, the use of auditory stimuli intrinsically limits the population tested (e.g., deaf participants can only be tested with visual stimuli). In the visual domain, a screen image that represents a dog or flower to human eyes will be off-color for most animals due to retinal differences in color vision and cone opsins (D’Eath, 1998). Letter shapes will be more familiar to adult literate humans than to infants or animals, while handshapes may be more meaningful to signers than non-signers.

In this study we introduce a new visual AGL paradigm to help resolve these empirical issues. We use stimuli made up of abstract visual tiles that can be presented sequentially (at differing rates) or simultaneously, granting us tight control over working memory issues. The tiles are non-representational, and thus equally unfamiliar to all participants (unlike the recorded speech syllables, letter strings, images, or animal calls used in most previous studies). As visual stimuli, they can be used to test deaf participants. Finally, and crucially, the visual elements can be flexibly arranged to examine all of the grammar types and complexity levels used in previous AGL studies, a capability we illustrate in the current study by testing all linguistically relevant levels of the formal language hierarchy [aka the extended “Chomsky hierarchy” (Jäger and Rogers, 2012)].

We exposed normal adults to grammatical sequences (“exposure phase”), and then examined the generalizations they made by presenting an assortment of novel test stimuli (“test phase”). Stimuli were generated with grammars at three key levels: a finite-state grammar, AB^N^A (Ravignani et al., 2013), possessing long-distance dependencies (where *N* indicates one or more Bs, so stimuli included “ABBA,” “ABBBBA,” etc.), a context-free “mirror” grammar, with nested dependencies (“AAB BAA”), and a mildly-context-sensitive “copy grammar” with crossed dependencies (“AAB AAB”).

Since Chomsky (1956) it has been widely recognized that human linguistic syntax requires computations that go beyond the simplest level of complexity (“regular” or “finite-state” grammars) in the formal language hierarchy. However, precisely what level supra-regular power linguistic syntax requires remained controversial until Shieber (1985) presented convincing empirical arguments that computations at the next complexity level, context-free grammars, are also not adequate to explain certain forms of crossing dependencies observed in natural languages, including Dutch and Swiss German. Computational linguists today agree that human syntactic competence must extend into the so-called “mildly context sensitive” level, that allows both nested and crossing dependencies (Stabler, 2004). In formal terms the mirror grammar (WW^R^) used here is located at the context free level (CFG) and the copy grammar (WW) at the mildly context-sensitive level (MCS) (Joshi et al., 1991; Jäger and Rogers, 2012).

Successful recognition of each grammar type requires the storage of at least one item in working memory, and our paradigm permits precise control over the length and complexity of the stimuli. We assessed mastery of the rules with two tests in our experiment: (a) whether participants could generalize to new numbers of syllables (*N* lower or higher than previously experienced) and (b) whether stimuli with missing elements (e.g., ABB AB) could be detected as erroneous.

All three grammars tested here contain at least one long-distance dependency (two dependent elements separated by at least one intervening element). In AB^N^A, the dependency exists solely between the first and last A, while in the other two grammars the number of dependencies is equal to the length of the first half of the sequence, and the corresponding dependencies in the second half are either nested or “mirrored” (in the case of WW^R^) or crossed (WW), and thus increase with *N*.

Both intuition and previous research suggest that crossing dependencies, as found in the copy grammar, although theoretically requiring more challenging computations, might be easier to process. For example, Bach et al. (1986) compared the abilities of Dutch and German speakers to process crossing and nested (center-embedded) dependencies, respectively, and documented an advantage for Dutch speakers, which they interpreted as reflecting a significant preference for the (Dutch-style) crossing dependency type for complex sentences. They interpreted these data as ruling out a push-down stack model of sentence processing (a push-down stack is the simplest computational mechanism that can generate and parse center-embedding). This prompted the introduction of embedded pushdown automata (EPDA, Joshi, 1990), which allow the processing of crossed dependencies using multiple embedded stacks. EPDAs can process both crossed and nested dependencies, but the latter are computationally more costly (in terms of numbers of items that have to be stored during computation). This would theoretically justify the intuition that crossed dependencies, despite being formally more complex, might nonetheless be processed more easily by humans, since load on working memory may be less than for nested dependencies. However, since these seminal experiments were based on natural language sentences, they had to leave other key variables (such as semantic content and lexical access) uncontrolled. While artificial stimuli do not have the richness of natural language, they allow key experimental variables to be more easily controlled, which is one of the reasons why they are predominantly used in AGL experiments. Both crossing and nested dependencies require the perceiver to maintain an item in memory while searching for its match later in the stimulus, and the number of remembered items increases with more dependencies. However, even simple finite-state grammars can have non-adjacent dependencies (Chomsky, 1956), so this “holding time” in working memory can and should be controlled in experiments seeking to understand structural processing.

Visual stimuli allowed us to minimize working memory load: in all conditions, abstract visual “tiles” appeared sequentially in both space and time and remained onscreen thereafter. Our stimuli also allow us to separate syntactic from semantic complexity, which were intertwined in Bach et al. (1986). By minimizing meaningfulness and titrating memory load we aimed to gain a clearer view of the syntactic processing differences between different types of long-distance dependencies independent of semantic interpretation.

## Materials and Methods

### Stimuli

Video stimuli in mp4 format were generated using Python (v. 2.7)^1^ and Quicktime (v. 7.6.6, Apple Inc.). Sequences consisted of small square non-representational elements that appeared sequentially on a black background at a rate of 6 frames per second, that is, a new element appeared every 166 milliseconds (ms), directly adjacent to the location of the previous element, so the entire sequence was completed one by one, akin to a word being typed. We also conducted an experiment (see Supplementary Material) where only one element was ever shown at a time, which posed a larger strain on working memory. Performance for this condition was very poor however, with failure to generalize to novel N for both copy and mirror grammars.

The individual elements were 20 × 20 pixels. In the middle of the sequence (for Copy and Mirror) or between As and Bs (for AB^N^A) a black rectangle (16 × 20 pixels) was presented, resulting in a temporal pause of 166 ms and a spatial gap of 16 pixels per black rectangle. Pilot work suggested that participants were not easily able to solve the Mirror and Copy tasks without such an overt marker of sequence structure (cf. Morgan and Newport, 1981).

**FIGURE 1:**
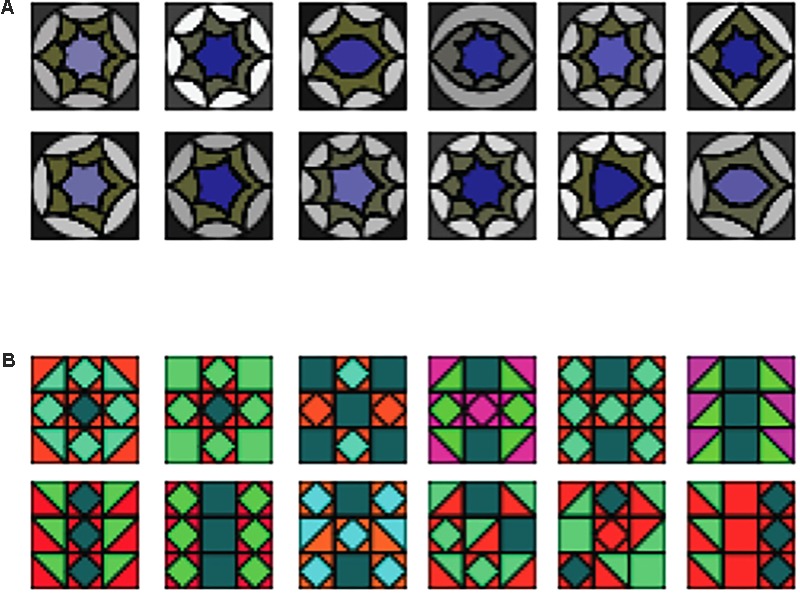
Examples of sequence elements (top rows: **(A)** elements, bottom rows: **(B)** elements).

**FIGURE 2:**
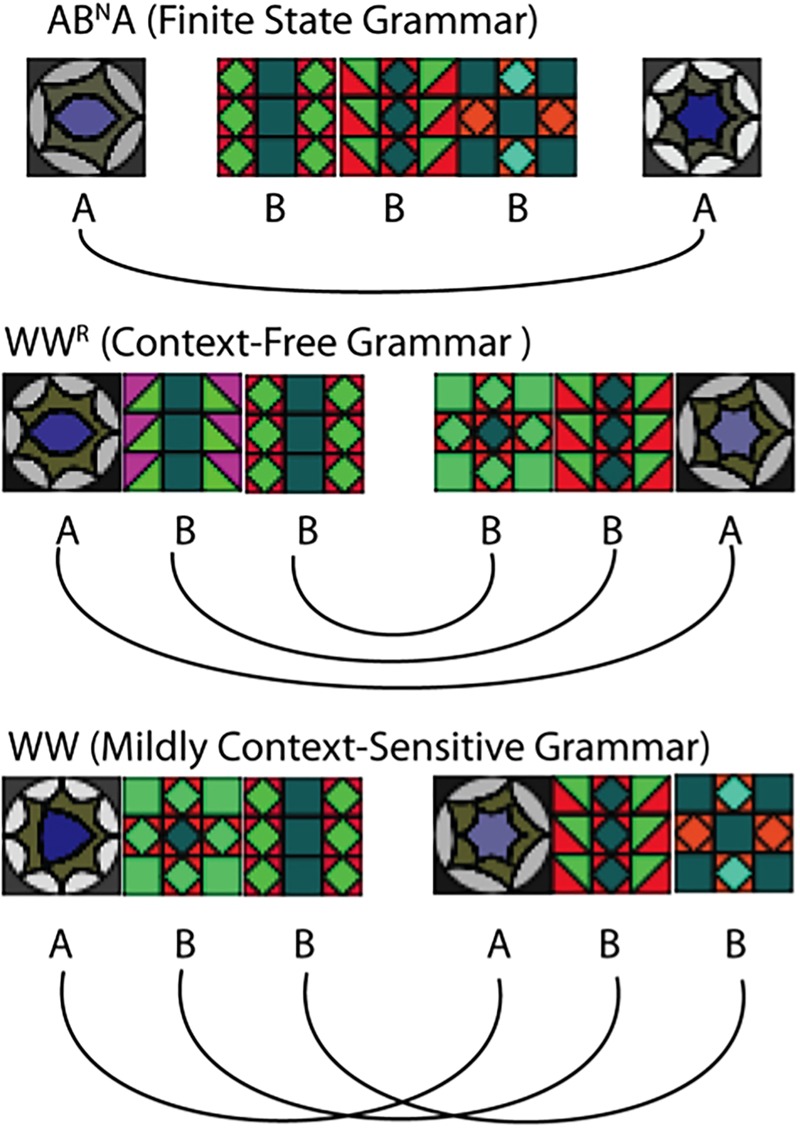
Examples of stimuli sequences for all three grammars with *N* = 3.

The twelve A tile elements (**Figure 1**) used a gray/purple color scheme and had rounded, nested shapes, while the twelve B elements were reddish and greenish and consisted of un-nested angular shapes [similar to stimuli used in (Stobbe et al., 2012)]. Similar nested shapes as in our A shapes have been used by Pothos and Bailey (2000), however, in our case the order within the nested shapes themselves was not meaningful. For each sequence, shapes were randomly chosen without replacement (**Figure 2** gives examples of complete sequences).

### Participants

We recruited 20 Italian speaking participants (*M*_age_ = 25.5 years, 14 females, 6 males) from the University of Milan-Bicocca community. One additional participant was excluded as he did not reach the training criterion (see below). Participants gave their written informed consent prior to taking part and received course credit. The study was approved by the ethics committee of the University of Milan-Bicocca (approval number Prot. 204).

### Procedure

#### Training

Prior to testing, participants completed a training session using simple AB^N^ sequences (used in many previous studies, Fitch and Hauser, 2004) to familiarize them with the procedure. They had to reach a criterion of at least 12 correct choices out of 15 in this training session (Exact binomial test, *p* = 0.02); participants could repeat the training twice. If they did not reach this criterion after three training runs, they were excluded from further testing (*N* = 1). Only during the warm-up grammar was the prompt *“Si o No”* (Yes or No) displayed after each sequence. Participants also received oral instructions: *“Ora il tuo compito è decidere se ogni nuovo video che vedi segue lo stesso schema di quelli della fase di familiarizzazione oppure no”* (Roughly: “Now your task is to decide if each new video that you see follows the same schema as those of the familiarization phase or not”).

#### Exposure

Each participant was tested on each grammar in random order. The exposure phase for each grammar lasted about 2 min. During the exposure phase participants saw 30 grammatical sequences with *N* = 2, *N* = 3, and *N* = 5. All participants saw the same sequences, but in a different randomized order.

#### Testing

In the test phase we showed individual sequences. After the final sequence element had appeared, the screen went blank (to discourage explicit counting or other slow conscious comparison strategies) and participants were prompted to indicate whether they thought the sequences followed the same schema as those they had seen during the exposure phase. Using a keyboard, participants pressed a key labeled with a green “*Si”* (Yes) if they thought the sequence followed the same rule, or a key marked with a red *“No”* if not. The response keys were C and N, and the placement of the stickers was counterbalanced across participants. Participants could not submit an answer before the sequence was completed. Response time was not limited and no feedback was given.

In total, there were 87 test stimuli for each grammar, summarized in **Table 1**. Twenty were novel exemplars of *N* = 2 and *N* = 3 stimuli. Participants were also tested on *N* = 4 and *N* = 6 sequences. *N* = 6 sequences required spontaneous generalization beyond the previously encountered *N*. *N* = 4 sequences were also novel to the participants, but did not require generalizing beyond the observed *N*s (suggested by Zuidema (2013) as a control for failure due to increased stimulus length].

**Table 1:** Summary of test stimuli.

Condition	AB^N^A	Mirror	Copy
Correct (*N* = 2,3)	20	20	20
Generalization (*N* = 4)	10	10	10
Generalization (*N* = 6)	6	6	6
Generalization with missing element (*N* = 4)	10	10	10
Generalization with missing element (*N* = 6)	6	6	6
Generalization with mismatched element (*N* = 4)	10	10	10
Missing element (*N* = 2,3)	10	10	10
Mismatched element (*N* = 2,3)	15	15	15
Total	87	87	87

The most critical test requires participants to distinguish valid extensions from extensions with missing elements (i.e., incomplete dependencies). Grammatical extensions can be accepted, and those with missing elements rejected, only if a generative form of the underlying grammar has been acquired. As distractor trials, we also included strings with the correct number of elements, but incorrect category members, for example ABB ABA.

### Analyses

Statistical analyses were conducted in the R statistical environment (v 3.3.3). For the Bayesian analysis, the consistency between participants’ responses and those expected for the hypothesized suite of possible grammars was assessed using Generalized Linear Mixed Models (GLMMs) fit with the ‘MCMCglmm’ package (Hadfield, 2010). Participants’ responses per trial were coded as being either consistent or inconsistent with the hypothesized grammars for each experimental session, and we subsequently fit univariate GLMMs for each set of trial-level responses (Bernoulli distribution, logit link function, average participant intercept term, and a participant-level random intercept effect). Model parameters were estimated using Markov Chain Monte Carlo (MCMC) sampling to facilitate unbiased estimation of marginal parameter densities, as well as to effectively quantify parameter uncertainty for hypothesis testing (Zhao et al., 2006). Weakly informative parameter expanded priors were specified for the participant random effect variance component to enhance MCMC mixing properties (Gelman, 2006), and diffuse normal priors were specified for the average participant intercept. Given that overdispersion cannot be estimated for repeated measures of a binary response variable (Skrondal and Rabe-Hesketh, 2007), the residual variance was fixed at 1 during model estimation. The posterior distribution of variance components and predicted intercepts were subsequently rescaled to approximate posterior distributions with 0 residual variance (Diggle et al., 2002). The size of the MCMC chain was adjusted to produce effective sizes of at least 1,000 samples for all intercept terms. To enhance the precision of parameter estimates, we did not thin MCMC chains (cf. Link and Eaton, 2012).

We first examined whether responses across the set of grammars were consistent with randomly consistent responses. If most participants induced the intended “target” grammar, then the target grammars would exhibit the highest probability of a consistent response, while most alternative grammars would be consistent with chance performance (50%). We then formed log odds ratios (*logORs*) comparing the logit-scale average participant intercept posteriors for the target grammar with that of each alternative model, again predicting that the target grammars would exhibit higher relative odds than the alternates. Log odds estimates were used for these analyses to correct for the skewness of the raw OR distribution.

After considering average performance across participants, we further investigated performance within each participant by calculating *logORs* between the predicted participant intercepts for each grammar model. For each target grammar, we considered an effect statistically significant if the 95% highest posterior density credibility interval (95% CI) for that grammar excluded alternative hypotheses. Note that the Bayesian GLMMs used here reduce the inferential risks of these multiple comparisons through partial pooling of the random effect estimates toward the average participant intercept (Gelman et al., 2012).

After comparing the target and alternative grammars across and within participants, we further assessed whether participants exhibited consistent individual differences in their ability to acquire and/or appropriately apply the target grammars. The degree of consistent among-participant variance in performance across trials was quantified using the intraclass correlation coefficient for the latent logistic distribution (Nakagawa and Schielzeth, 2010). We then further investigated the degree to which consistent responses for the three target grammars correlated across participants by specifying a multi-response GLMM with grammar-specific intercepts and unstructured covariance between the participant identity random effects. If positive correlations are detected across the experimental sessions, among-participant variance for the target grammars may reflect individual differences in some more generalized aspect of their performance. A more detailed theoretical overview of our statistical analyses with accompanying R code and a brief tutorial are provided in the Electronic Supplementary Material (ESM) to assist other researchers interested in applying our methods in future AGL studies. Our data, stimuli and code are available on request from the senior author (WTF).

## Results

### Overall Performance

We first analyzed basic metrics of successful grammar acquisition. For each grammar, we used Wilcoxon signed rank tests to compare the responses to grammatical and ungrammatical stimuli (the latter violating the experimenters’ intended grammar). At the group level, we analyzed the percentage of “same” responses (i.e., the participant found it congruent with the exposure stimuli) for grammatical and ungrammatical stimuli. For AB^N^A, “same” responses were made to 94.3% of grammatical and only 14.6% of ungrammatical strings (*W* = 400, *p* < 0.001, effect size: Cohen’s *d* = 4.97). For the mirror grammar, participants responded “same” to 90.7% of grammatical and 19.5% of ungrammatical strings (*W* = 397, *p* < 0.001, effect size *d* = 4). For the copy grammar, “same” responses were given to 91.8% of grammatical and 26.9% of ungrammatical strings (*W* = 397, *p* < 0.001, effect size *d* = 3.5). Thus, overall, participants successfully rejected ungrammatical foils and accepted novel grammatical strings for all three grammars. These results show that participants learned to recognize the exposure pattern, and were able to generalize beyond the previously seen exposure sequences to novel sequences.

**FIGURE 3:**
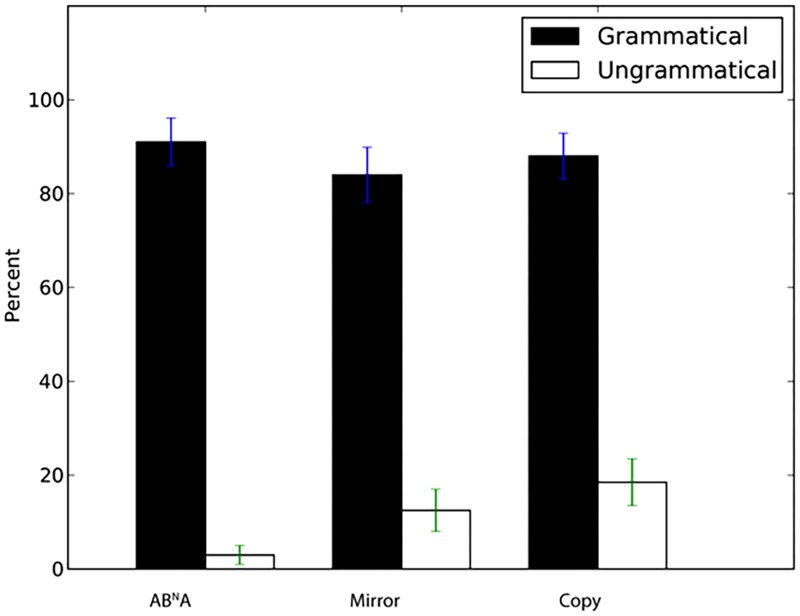
Percentage of “same” response (±SEM) for novel grammatical *N* = 4 sequences and ungrammatical sequences with incomplete dependencies.

We next examined generalization to novel sequences with lengths different from those in the training set. Tile number was *N* = 2, 3, and 5 in the exposure stimuli (yielding total string length of *N* + 2 for AB^N^A and *N*^∗^2 for Mirror and Copy, e.g., for *N* = 3 sequences for AB^N^A total length was 5, while it was 6 for Mirror and Copy). Participants again showed a significantly higher percentage of “same” answers to grammatical *N* = 4 sequences than for incorrect “foil” sequences with incomplete dependencies (**Figure 3**, AB^N^A: *W* = 388.5, *p* < 0.001, *d*’ = 2.88, Mirror: *W* = 378, *p* < 0.001, *d*’ = 2.29, Copy: *W* = 385, *p* < 0.001, *d*’ = 2.23). Thus, participants readily generalized to stimulus lengths intermediate between those observed in exposure, and rejected ill-formed incomplete stimuli.

**FIGURE 4:**
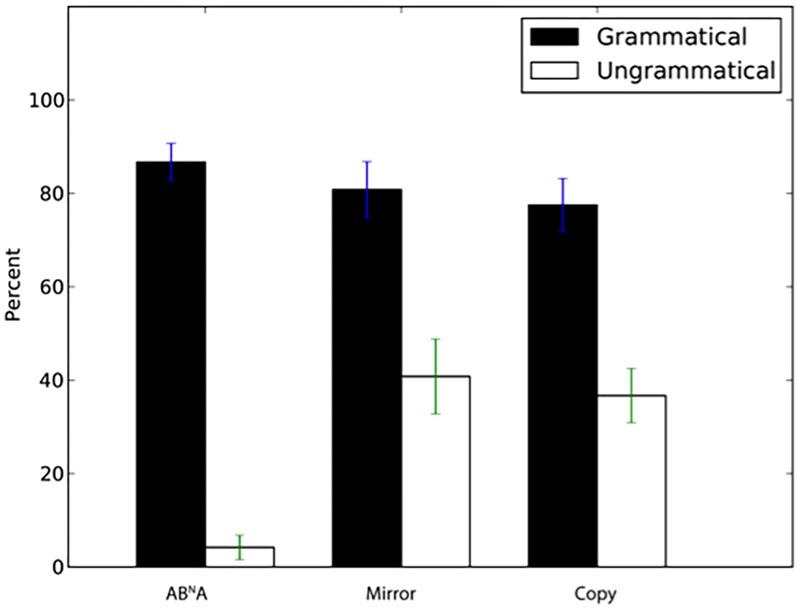
Percentage of “same” responses (±SEM) for novel grammatical *N* = 6 sequences and ungrammatical sequences with incomplete dependencies.

Participants also had a significantly higher percentage of correct “same” answers to novel *N* = 6 sequences than for sequences with incomplete dependencies (**Figure 4**, AB^N^A: *W* = 398, *p* < 0.001, *d*’ = 2.32, Mirror: *W* = 313.5, *p* = 0.002, *d*’ = 1.1, Copy: *W* = 344, *p* < 0.001, *d*’ = 1.07).

### Individual Performance

We next analyzed individual performance by asking, for each participant, whether their number of “correct” answers exceeded a criterion value (determined by the minimal number to achieve a binomial test *p* < 0.05; this actual *p* varied from 0.01 to 0.049 depending upon the sample size). **Table 2** gives the number of participants who exceeded this criterion, and shows that all participants for all grammars could distinguish grammatical from ungrammatical stimuli that were length *N* = 2 and 3 (the lengths encountered during training). For AB^N^A and copy grammars, most participants successfully generalized to *N* = 4 (19/20 and 16/20, respectively), while only 7/20 did so for the mirror grammar. Generalization to *N* = 6 (beyond the previously encountered sequence lengths) occurred only for about a third of participants for copy and mirror grammars (8/20 and 7/20, respectively), while individual performance remained high for AB^N^A for *N* = 6 (18/20). Thus, although all individuals detected the underlying pattern and could apply it to same-length stimuli, there was considerable variability in the degree to which participants generalized beyond these lengths.

**Table 2:** Individual participant performance on novel stimuli in the test phase (number of participants successful, out of 20 participants total for each grammar).

Condition	AB^N^A	Mirror	Copy
*N* = 2,3 and mismatches	20 (100%)	20 (100%)	20 (100%)
*N* = 4 and mismatches	19 (95%)	7 (35%)	16 (80%)
*N* = 6 and mismatches	18 (90%)	7 (35%)	8 (40%)

*Success was evaluated by the following criteria: Grammatical *N* = 2 and 3 sequences and incomplete dependencies (at least 20/30 trials correct, binomial *p* = 0.049). Grammatical *N* = 4 sequences and incomplete dependencies (at least 15/20 correct, *p* = 0.02). Grammatical *N* = 6 and sequences and incomplete dependencies (at least 10/12 correct, *p* = 0.02). Strings of the copy grammar consisted of a repetition of the first half of the string (AABAAB), while strings of the mirror grammar contained a repetition of the first half in reversed order (AABBAA). AB^*N*^A strings began and ended with an A, with a varying number of B in the middle (ABBBA).*

Since each participant was tested on each grammar, but in different orders, we also tested whether the sequence of testing had an effect. There was a significant difference between the performance for sessions run first, second or third only for the mirror grammar (Kruskal–Wallis test; χ^2^ = 6.46, df = 2, *p* = 0.04), but *post hoc* pairwise comparisons did not reach significance for Bonferroni-corrected *p*-values. This suggests that prior experience with one type of sequence pattern does not greatly influence the processing of other types of patterns.

Performance declined significantly as stimulus length increased for multi-dependency Copy and Mirror strings, but not for AB^N^A strings. We found no performance differences between the string lengths for AB^N^A (Kruskal-Wallis rank sum test, χ^2^ = 2.85, df = 2, *p* = 0.24). Strings with missing elements in the mirror and copy conditions had lengths 3, 5, 7, and 11, while the lengths for AB^N^A were 3, 5, and 7. For the mirror and copy grammars we found significant differences among the string lengths (χ^2^ > 12.66, *p* < 0.005). For the mirror grammar, the significant differences lay between lengths 3/4 and 11/12 (*W* = 316.5, *p* = 0.001, effect size *d* = 5.75) and 5/6 and 11/12 (*W* = 327, *p* = < 0.001, effect size *d* = 5.72). For the copy grammar, the significant difference lay between lengths 3/4 and 11/12 (*W* = 318.5, *p* = 0.001, Bonferroni-corrected α = 0.008, Effect size *d* = 5.01). Performance in the distracter task (sequence had the correct number of tiles, but one tile had the wrong category membership) is summarized in **Table 3**.

**Table 3:** Individual performance (number successful, out of 20 participants total) on the distracter task (sequence with a single incorrect tile) for *N* = 2, 3, and 4.

Condition	AB^N^A	Mirror	Copy
*N* = 2,3	14 (70%)	15 (75%)	11 (75%)
*N* = 4	14 (70%)	11 (75%)	11 (75%)

*Success criterion was at least 9/10 correct (*N* = 4, binomial *p* = 0.01) or 12/15 correct (*N* = 2, 3, *p* = 0.02).*

### Bayesian Model Selection Analysis

A key issue in AGL is that participants may show above-chance performance, but nonetheless have induced a grammar different from the exact intended “target” grammar the experimenters used to generate the stimuli (van Heijningen et al., 2009; Fitch and Friederici, 2012; Ravignani et al., 2015). This would be particularly problematic if an alternate grammar at a lower level of grammatical complexity could be adopted and still yield “successful” performance, as observed in previous experiments with animals (van Heijningen et al., 2009; Ravignani et al., 2015). To evaluate this possibility we analyzed our data using a multilevel Bayesian modeling framework, implemented in R (see ESM for a detailed description of our statistical framework). The consistency of responses for each particular test string with a large set of possible alternative grammars was first computed for each participant (for a description of this alternate set see the ESM). The probability of each of these possible grammars being the one adopted by that participant could then be computed, using Monte Carlo Markov Chain (MCMC) simulations to fit parameters and quantify uncertainty in parameter values. Finally, we computed the odds ratio, relative to our intended target grammar, for each of these possible alternatives. Because of skewness, the natural log odds ratio (base *e* log, “*logOR*” hereafter) is presented (so *logOR* of 2 means the odds of utilizing the target grammar are *e*^2^ or 7.4 times larger than the alternative).

We first examined whether responses across the set of grammars were consistent with random responses. If most participants induced the intended “target” grammar, then the target grammars would exhibit the highest probability of a consistent response per trial, while most alternative grammars would be consistent with chance performance (*p* = 0.50). If there is no overlap between the 95% credibility intervals for the target grammar and any of the other grammars, we can confidently conclude that the participants’ behavior was significantly more likely to be consistent with that target relative to any of the alternatives. We further estimated the posterior probability of utilizing each grammar by quantifying the proportion of MCMC samples that overlapped alternative hypotheses (hereafter *p*_MCMC_). In particular, *p*_MCMC_ represents the proportion of posterior samples ≤ 0.50 for the probability of a consistent response and ≤0 for the target to alternative grammar logOR. In the case that an alternative grammar on average received stronger posterior support than the target, we computed the proportion of samples where logOR ≥ 0 as a measure of support for the alternative grammar.

**FIGURE 5:**
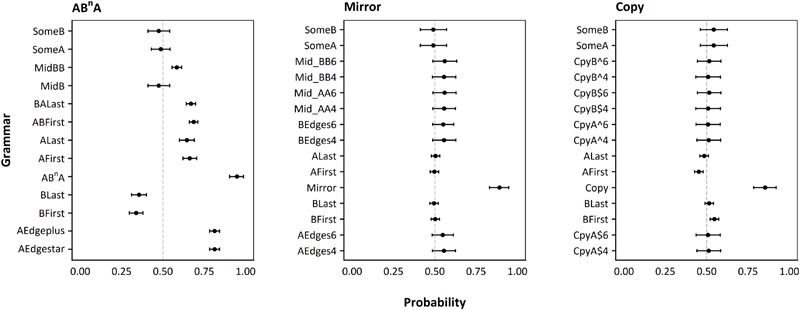
Probability of each alternative grammar. Each dot represents the mean probability of the grammar given on the *y* axis given all participants’ responses combined, with error bars representing 95% credibility intervals, as calculated by Monte Carlo Markov Chain simulations in a Bayesian framework (see text for details). The line at 50% shows the success expected by chance in our two-alternative forced-choice paradigm. Left panel: Results for AB^N^A grammar (finite-state). Middle panel: Results for mirror grammar (context-free). Right panel: Results for copy grammar (mildly context-sensitive).

Average participant responses provided strong support for successful induction of the target grammars (see **Figure 5**). As predicted, high probabilities of success were found for the copy (*M*_p_ = 0.85, 95% CI [0.78, 0.92], *p*_MCMC_ < 0.001), mirror (*M*_p_ = 0.89, 95% CI [0.83, 0.95], *p*_MCMC_ < 0.001), and AB^N^A (*M*_p_ = 0.95, 95% CI [0.90, 0.99], *p*_MCMC_ < 0.001) target grammars. For the mirror session, all of the alternative grammars were consistent with random responses. For copy, the BFirst grammar exhibited a low but above chance probability of success (*M* = 0.55, 95% CI [0.52, 0.57], *p*_MCMC_ < 0.001). Nonetheless, the odds of the average participant responding consistently with the copy grammar are 5 times larger than the odds for BFirst (*M*_LogOR_ = 1.61, 95% CI [1.03, 2.18], *p*_MCMC_ < 0.001; see ESM Figure S4.1). In AB^N^A, many of the alternative grammars exhibited above chance probabilities of success, but the *logOR*s all suggested significantly higher odds for AB^N^A (ranging from AEdge^∗^ and AEdge + : M_LogOR_ = 1.54, 95% CI [0.60, 2.54], *p*_MCMC_ < 0.001; to BFirst: M_LogOR_ = 3.68, 95% CI [2.75, 4.68], *p*_MCMC_ < 0.001), see ESM Figure S4.1.

### Individual Participant Model Selection Analysis

After considering average performance across participants, we further investigated individual performance (within participant) by calculating *logOR* between the predicted participant intercepts for each grammar model. Again, for each target grammar, we computed *p*_MCMC_ values and considered an effect statistically significant if the 95% highest posterior density credibility interval (95% CI) for that grammar excluded all alternative hypotheses. For both copy and mirror, we found that the majority of participants were significantly more likely to have utilized the target rather than alternative grammars, although some participants exhibited marginally lower differences in their odds of using the target or an alternative grammar (see ESM Figures S4.2 and S4.3). For AB^N^A, however, we could not exclude the possibility that some participants induced a different finite-state grammar, as five participants’ responses were significantly more consistent with the AEdge+ and AEdge^∗^ alternative grammars (*p*_MCMC_ < 0.05; see ESM Figure S4.4), which require As at the beginning and end, but ignore anything in between (this could be empty, or contain any other combination of As or Bs).

Finally, after comparing the target and alternative grammars between and within participants, we further assessed whether participants exhibited consistent individual differences in their ability to acquire and/or appropriately apply the target grammars. The degree of consistent among-participant variance in performance across grammars was quantified using the intraclass correlation coefficient. Participants exhibited consistent between-individual differences across the copy (*ICC* = 0.30, 95% CI [0.16, 0.46]), mirror (*ICC* = 0.34, 95% CI [0.17, 0.53]), and AB^N^A (*ICC* = 0.50, 95% CI [0.30, 0.72]) target grammars. We then utilized a multi-response model to quantify correlations in participants’ performance across target grammars, and we calculated *p*_MCMC_ as the proportion of posterior correlation estimates ≤ 0. We found relatively high correlations, indicating that some participants were consistently better than others across all grammars. Because our participants performed no tasks other than pattern perception, we cannot determine whether these consistent individual differences reflect some general motivational or attentional variable, or a cognitive mechanism more specific to pattern perception.

Among-participant correlation between copy and mirror grammars was 0.47 (0.08, 0.81)^∗^, 0.59 (0.25, 0.89)^∗∗^ between copy and AB^N^A and 0.52 (0.15, 0.85)^∗^ between mirror and AB^N^A (^∗^ = *p*_MCMC_ < 0.05; ^∗∗^ = *p*_MCMC_ < 0.01). We report posterior correlations with corresponding 95% highest posterior density credibility intervals. Correlations were derived from the estimated covariance between participant random effects across the three target grammars in a multi-response multilevel model.

## Discussion

To forestall confusion, note that we use the term “grammar” here in a specific technical manner, to indicate any rule system that generates stimuli possessing some type of pattern. This is consistent with a voluminous literature on AGL dating back to Arthur Reber’s work (Reber, 1967) where use of the term “grammar” by itself carries no implications about the relevance of the rule system to human language. Furthermore, the type of items used to construct stimuli, whether visual, auditory, or tactile, are irrelevant to the underlying patterns that are investigated in these experiments, and a series of ones and zeros could be used without loss of generality (Nowak et al., 2002): the theory remains the same and the same descriptive machinery applies. One could perform the same experiment with images of cats and dogs, following Saffran et al. (1996), and the same principles would apply (though in that case it would be more likely that participants relied on verbal categorization, e.g., “cat, dog, dog, cat”). Second, while we can be certain about the precise grammar (set of rules) we used to generate a set of stimuli, we can never be certain what participants exposed to these stimuli take away: this is the empirical question we seek to answer, and the answer may well vary from participant to participant. We thus use the phrase “learn a grammar” in this precise technical sense: that after exposure to a set of well-formed exemplars, participants are able to correctly reject novel stimuli violating the rules of the experimental grammar, and correctly accept novel stimuli conforming to it, at a level significantly exceeding that expected by chance or predicted by alternative models. To yield convincing results, the set of “correct” stimuli tested must include novel stimuli differing in length from the exposure stimuli (to avoid template matching), while incorrect stimuli (“foils”) should include items designed to be consistent with various alternative grammars the participants might have induced (Fitch and Friederici, 2012).

In the present AGL paradigm, based on visual stimuli, our participants rapidly learned grammars of varying complexity levels after exposure only to grammatical exemplars and without explicit feedback. For the simple finite state grammar, AB^N^A, generalization occurred for lengths higher than the exposure *N*. For the two supra-regular grammars, performance was less impressive: for the copy grammar, the majority of participants were able to generalize to novel *N* beyond those encountered during exposure, while this was only true for a third of participants with the mirror grammar. Thus, we found a slight advantage for the copy grammar over the mirror grammar for *N* = 4. This is in agreement with, for example, a processing advantage for crossing over nested dependencies in Chinese tones (Li et al., 2013). However, in our study no advantage for crossing dependencies was observed for strings of length *N* = 6, where a similar proportion of participants was successful in mirror and copy grammars. This is partially consistent with Bach et al. (1986), who found an advantage for crossed over nested dependencies for three (but not two) levels of embedding, as well as with the processing advantage for crossed dependencies that de Vries et al. (2012) report for strings with three (but not two) dependencies. The somewhat puzzling inconsistency is that these studies found an advantage for crossing dependencies for longer strings, while we did so for shorter strings.

Furthermore, such a processing advantage for crossed dependencies is not reported by all studies. Uddén and colleagues, for example, reported no qualitative differences in performance for crossed and nested dependencies using visual sequences after acquisition of the basic rule (Uddén et al., 2012). Similarly Öttl and colleagues found no significant performance differences while processing crossing and nested dependencies (Öttl et al., 2015) in the auditory domain. Neither of these studies tested for generalization to new *N*. Thus, the processing advantage for crossing dependencies seems to be slight and does not manifest itself in all published experimental setups. The lack of any advantage for mirror or copy grammar with *N* = 6 in our data may result from working memory constraints specific to longer sequences being alleviated in our visual stimuli. One reason for this inconsistency might be that our stimuli consisted of novel abstract shapes while Bach et al. used actual meaningful sentences and de Vries et al. (2012) used spoken syllables, which would both have been highly familiar to the hearer. The processing load may thus have been higher in our study, with a concomitant decrease in performance for longer strings.

In our experiment, failure with higher *N* seems unlikely to result solely from memory constraints or stimulus length, since performance for AB^N^A remained high even for the longest sequences. Instead, we suggest that the failure at higher *N* is a result of the increasing number of dependencies that must be processed for the copy and mirror grammars, in contrast to AB^N^A. There appears to be a critical difference between four and six dependencies in these two grammars that may reflect an upper limit for the number of dependencies that can be processed under these conditions (rather than total number of items, since AB^N^A is not affected). At length *N* = 4, and thus within the posited upper limit of dependency processing, we found an advantage of Copy over Mirror strings, possibly due to mechanisms akin to embedded pushdown automata. This advantage seems to disappear for longer strings, however, where such mechanisms may be overtaxed by the number of dependencies.

The underlying intent of our experiment is that participants processed the strings incrementally rather than all at once, which was encouraged by the sequential presentation modality. Consistent with this intent, if the strings had been processed all at once, Mirror strings should have been processed more accurately due to their mirror symmetric structure (Wagemans, 1997; Treder, 2010), but this was not the case. Furthermore, we found no significant effect of session order on performance, suggesting that brief exposure to different grammars does not yield improvements in performance on others, across a single 1-h session.

One open issue concerns our use of a center marker in the two symmetrical grammars (mirror and copy grammars). This intuitively makes the online task easier, in the sense that a viewer can recognize immediately when the center of a stimulus has been reached, and indeed our pilot data suggested that participants found the task more challenging without such a marker. Of course, once the entire stimulus is visible, it is relatively trivial to identify the center point visually, so we suspect that participants would still be able to learn these grammars based on unmarked strings.

From a theoretical viewpoint having a center marker in our stimuli does not change the level of formal power needed to learn our different grammars, but it does change the precise nature of the automaton corresponding to that formal level. Specifically, unmarked strings require non-deterministic automata, while with center-marked strings the automaton can be deterministic (Parkes, 2002). The non-deterministic version must entertain multiple possibilities about where the center point might be, in parallel, and thus intuitively seems more difficult (unless implemented on a parallel processing machine). This possibility could be examined empirically in future research by directly comparing the time required to master marked vs. unmarked strings. However, because the current study compares deterministic pushdown automata (for the context-free grammar) with deterministic embedded pushdown automata (for mildly context-sensitive grammar), the presence or absence of center markers does not affect our main conclusion: that participants in our study were able to master grammars at all three levels of the formal language hierarchy.

In summary, those of our participants who were successful with large *N* spontaneously generalized to lengths greater than those encountered during exposure for mirror and copy grammars, showing for the first time that it is possible to learn and generalize complex visual grammars (context-free and mildly-context sensitive grammars) after only a few minutes of exposure. Success in our visual paradigm occurred without any explicit instructions about the nature of the sequence (e.g., instructions to notice or count the dependencies), and was independent of any semantic content or interpretation, which were absent in our stimuli. It is widely agreed that phrasal syntax in natural language requires processing at the mildly-context sensitive level; Jäger and Rogers, 2012), although other components of language such as phonology have been argued to require only regular (finite-state) level processing (Heinz and Idsardi, 2011, 2013). Participants in the current study successfully mastered rule systems at both of these levels of complexity in the non-linguistic domain of vision, specifically using abstract visual tiles (rather than words, syllables or phonemes). In the terms of formal language theory, these results suggest that the pattern-processing mechanisms in the visual processing domain attain equivalent computational power to those used to process spoken or written language. Although it remains unknown whether the same specific neural mechanisms are deployed across these different domains, our results suggest that human pattern-processing capabilities extend across multiple sensory domains, and thus are properties of the human mind in general and not limited to language processing. These results suggest that pattern-processing mechanisms are available in the visual processing domain, with equivalent computational power as those underlying the perception and production of spoken language.

## Outlook

The current study opens a conceptual door to further research in at least three domains. The first is to extend these results to nonhuman animals, as already attempted with simpler grammars in several previous studies (Stobbe et al., 2012; Ravignani et al., 2013; Sonnweber et al., 2015). These studies demonstrated that various animal species, including pigeons, keas, and chimpanzees, are able to process visual patterns at the finite-state level, but found no evidence for supra-regular processing. The stimuli and paradigm tested here would support further research in this direction. The second would be to investigate the neural basis for complex grammar processing, both in patients (e.g., Broca’s aphasics) or in brain imaging studies (e.g., fMRI). While there is considerable work on patients using simple finite-state grammars (e.g., Knowlton and Squire, 1996) and a large body of neurolinguistic work with more complex grammars using specifically linguistic stimuli (e.g., (Friederici et al., 2002; Hagoort, 2005; Pallier et al., 2011), there is little work examining the neural basis of supra-regular grammar processing in non-verbal domains. The final, and to us most exciting, research direction would be to examine the development of pattern-processing abilities across the life-span (e.g., in pre-verbal infants and young children) and in atypical populations (e.g., in deaf individuals). In particular, deaf signers, whose default mode of linguistic communication is visual, would be a fascinating population to study in this context. It seems plausible that their greatly increased experience with complex visual sequences relative to hearing individuals (Geraci et al., 2008) might lead to increased performance with our complex visual grammars.

## Ethics Statement

This study was carried out in accordance with the recommendations of the ethics board of the University of Milan-Bicocca. The protocol was approved by the ethics board of the University of Milan-Bicocca. All subjects gave written informed consent in accordance with the Declaration of Helsinki.

## Author Contributions

GW-F, BG, CC, and WTF designed the study. BG gathered the data. GW-F, JM, and WTF analyzed the data. GW-F and WTF wrote the manuscript.

## Conflict of Interest Statement

The authors declare that the research was conducted in the absence of any commercial or financial relationships that could be construed as a potential conflict of interest.

## Footnotes

1 www.python.org
