# Supplementary material for: Artificial Grammar Learning Capabilities in an Abstract Visual Task Match Requirements for Linguistic Syntax
Source: Front Psychol. 2018 Jul 24;9:1210. doi: 10.3389/fpsyg.2018.01210 (PMC6066649; doi:10.3389/fpsyg.2018.01210)
Supplement: Supplementary file 1 [file Table_1.DOC]

| Symbol | Explanation |
| --- | --- |
| . | any single character |
| .* | 0 or more characters |
| .+ | 1 or more characters |
| ^ | string beginning |
| $ | string end |

| COPY – Alternate Rule | Regular Expression | Description |
| --- | --- | --- |
| SomeA | .*A.* | any string that contains A |
| SomeB | .*B.* | any string that contains B |
| CpyB^6 | ^B..B..$ | B--B-- |
| CpyB^4 | ^B.B.$ | B-B- |
| CpyB$6 | ^..B..B$ | --B--B |
| CpyB$4 | ^.B.B$ | -B-B |
| CpyA^6 | ^A..A..$ | A--A-- |
| CpyA^4 | ^A.A.$ | A-A- |
| ALast | .*A$ | string ends with A |
| AFirst | ^A.* | string starts with A |
| BLast | .*B$ | string ends with B |
| BFirst | ^B.* | string starts with B |
| CpyA$6 | ^..A..A$ | --A--A |
| CpyA$4 | ^.A.A$ | -A-A |

| MIRROR-Alternate Rule | Regular Expression | Description |
| --- | --- | --- |
| SomeB | .*B.* | any string that contains B |
| SomeA | .*A.* | any string that contains A |
| Mid_BB6 | ^..BB..$ | --BB-- |
| Mid_BB4 | ^.BB.$ | -BB- |
| Mid_AA6 | ^..AA..$ | --AA-- |
| Mid_AA4 | ^.AA.$ | -AA- |
| BEdges6 | ^B....B$ | B----B |
| BEdges4 | ^B..B$ | B--B |
| ALast | .*A$ | any string that ends with A |
| AFirst | ^A.* | any string that starts with A |
| BLast | .*B$ | any string that ends with B |
| BFirst | ^B.* | any string that starts with B |
| AEdges6 | ^A....A$ | A----A |
| AEdges4 | ^A..A$ | A--A |

| AB^N^A-Alternate Rule | Regular Expression | Description |
| --- | --- | --- |
| SomeB | .*B.* | any string that contains B |
| SomeA | .*A.* | any string that contains A |
| Mid_BB | .+BB.+ | contains BB, not at the edge |
| Mid_B | .+B.+ | contains B, not at the edge |
| BALast | .*BA$ | string ends with BA |
| ABFirst | ^AB.* | string starts with AB |
| ALast | .*A$ | string ends with A |
| AFirst | ^A.* | string starts with A |
| BLast | .*B$ | string ends with B |
| BFirst | ^B.* | string starts with B |
| AEdge+ | ^A.+A$ | A on edges, with something in between |
| AEdge* | ^A.*A$ | A on edges, possibly nothing else |
