# Supplementary material for: Artificial Grammar Learning Capabilities in an Abstract Visual Task Match Requirements for Linguistic Syntax
Source: Front Psychol. 2018 Jul 24;9:1210. doi: 10.3389/fpsyg.2018.01210 (PMC6066649; doi:10.3389/fpsyg.2018.01210)
Supplement: Supplementary file 2 [file Table_2.DOCX]

**Table S1.** Intraclass correlation coefficients for target grammar performance

|  | Copy | Mirror | AB^n^A |
| --- | --- | --- | --- |
| *ICC* (95 % CI) | 0.30 (0.16, 0.46) | 0.34 (0.17, 0.53) | 0.51 (0.30, 0.72) |

*Note*. Latent-scale intraclass correlation coefficients (*ICC*s) are reported with corresponding 95% highest posterior density credibility intervals. Posterior samples were scaled to approximate parameters from a joint distribution with no residual variance, and the logistic distribution variance $\pi^{2}/3$ was subsequently used for the latent-scale residual variance in all ICC calculations. See the electronic supplementary material for a detailed description of this calculation.

**Table S2.** Among-subject correlations between target grammar performance

|  | Copy | Mirror | AB^n^A |
| --- | --- | --- | --- |
| Copy | -- |  |  |
| Mirror | 0.47 (0.08, 0.81) | -- |  |
| AB^n^A | 0.59 (0.25, 0.89) | 0.52 (0.15, 0.85) | -- |

*Note*. Posterior correlations with corresponding 95% highest posterior density credibility intervals are reported. Correlations were derived from the estimated covariance between subject random effects across the three target grammars in a multivariate mixed effects model.
