# Supplementary material for: Artificial Grammar Learning Capabilities in an Abstract Visual Task Match Requirements for Linguistic Syntax
Source: Front Psychol. 2018 Jul 24;9:1210. doi: 10.3389/fpsyg.2018.01210 (PMC6066649; doi:10.3389/fpsyg.2018.01210)
Supplement: Supplementary file 3 [file Table_3.docx]

**Electronic Supplementary Material for:**

[Artificial grammar learning capabilities in a visual task match requirements for linguistic syntax](http://review.frontiersin.org/Document/DownloadPDF?articleId=387357&siteId=39&userId=296814&roleId=16)

**Gesche Westphal-Fitch^a,b^, Beatrice Giustolisi^c^, Carlo Cecchetto^d^, Jordan S. Martin^a,e^, and W. Tecumseh Fitch*^a^**

**^a^ University of Vienna, Department of Cognitive Biology, Althanstrasse 14,**

**1090 Vienna, Austria**

**^b^ Medical University of Vienna, Department of Neurology, Währinger Gürtel 18-20, 1090 Vienna, Austria**

**^c^ University of Milan-Bicocca, Department of Psychology, Piazza dell’Ateneo Nuovo 1, 20126 Milan, Italy**

**^d^ Structures Formelles du Langage (Unité Mixte de Recherche CNRS & Université Paris 8), 59 rue Pouchet, 75017 Paris**

**^e^Department of Anthropology, Emory University, Atlanta, Georgia**

**Contents Page**

**Introduction** 2

**Text S1**. Theoretical motivation 2

**Text S2.** Fitting the univariate GLMM 7

**Text S3.** Subject specific estimates 13

**Text S4.** Fitting the multivariate GLMM 15

**Introduction**

Here we present a theoretical overview and brief tutorial following the statistical analyses reported in the main paper. Example R code and accompanying description are provided to assist other researchers in applying Bayesian generalized linear mixed modeling (GLMM) for both average and subject specific hypothesis testing in artificial grammar learning experiments. Basic familiarity with Bayesian modeling and the R coding language is assumed herein. We therefore do not discuss R syntax, data preparation, and model diagnostics in detail. In addition to the references provided in the main text, detailed course notes for fitting Bayesian regression models in the R package ‘MCMCglmm’ (Hadfield, 2010) are available online at the following [link](ftp://cran.r-project.org/pub/R/web/packages/MCMCglmm/vignettes/CourseNotes.pdf). Note that equations in **Text S1** are referenced throughout the tutorial.

**Text S1.** Theoretical motivation

In the present paper, we were interested in estimating the odds of subjects’ utilizing either the target grammar or alternative grammars during an experimental trial. We therefore coded whether trial responses were consistent (‘1’) or inconsistent (‘0’) with each of the hypothesized grammar rules. For example, rejecting the string ‘ABAB’ would be consistent with a subject’s use of the **AB^n^A** rule, but would be inconsistent with use of the **(AB)^n^** rule. Marginalizing over the effects of subject identity, trial responses within each experimental session can be represented as random samples from the Bernoulli random variable

$$Y_{g} \sim Ber(p_{g})$$

for hypothesized grammar$g (g=1,\ldots,k)$ with the following distribution

$$P\left( Y_{g}=1 \right)=E\left( Y_{g} \right)=p_{g}$$

$$P\left( Y_{g}=0 \right)=1-p_{g}$$

such that $p_{g}$ is the probability of correctly classifying a stimulus as consistent or inconsistent with grammar $g$. Note that the binomial distribution reduces to the Bernoulli distribution for the repeated trial-level sampling considered here.

Although subjects were presented with a binary choice for each trial, we considered these responses to reflect continuous rather than discrete variation in grammar rule acquisition and application. A generalized linear model can be specified for $Y_{g}$ to estimate the latent, or unobserved, continuous random variable $Y_{g}^{*}$. Here we consider an intercept-only logistic regression model of the observed trial $i$response $(i=1,\ldots,n)$ for grammar $g$ with residual error $\varepsilon_{gi}$

|  | $y_{gi}^{*}=\alpha_{g}+\varepsilon_{gi}$ | (1) |
| --- | --- | --- |

$$\alpha_{g}=logit\left( \frac{p_{g}}{1-p_{g}} \right)$$

The logit function links $Y_{g}$ to the latent random variable $Y_{g}^{*}$ (Skrondal & Rabe-Hesketh, 2004) such that

$$y_{gi}=\left\{ \begin{matrix} 1 & y_{gi}^{*}>0 \\ 0 & otherwise \end{matrix} \right.$$

For the remainder of the text, we exclude the $i$ subscript for notational simplicity. The estimated probability of a grammar consistent response $\hat{p}_{g}$ can be calculated from the estimated log odds of a consistent response $\hat{\alpha}_{g}$ by the logistic function

|  | $\hat{p}_{g}=logistic\left( \hat{\alpha}_{g} \right)=\frac{e^{\hat{\alpha}_{g}}}{{1+e}^{\hat{\alpha}_{g}}}$ | (2) |
| --- | --- | --- |

Note that residual error $\varepsilon_{g}$ and its variance $\sigma_{g\varepsilon}^{2}$ cannot be meaningfully estimated for (1) because the relationship between the sample mean and variance for observations of Bernoulli random variable $Y_{g}$ is fixed, such that

$$Var\left( y_{g} \right)=\bar{y}_{g}\left( 1-\bar{y}_{g} \right)\approx\hat{p}_{g}\left( 1-\hat{p}_{g} \right)$$

This result generalizes to multilevel, or mixed effects, models and precludes the possibility of overdispersion in (1) resulting from unmodeled covariates (Skrondal & Rabe-Hesketh, 2007). During model estimation, the residual variance of (1) should therefore be fixed to a constant value, and herein we assume unit variance for the estimated residual error ($\hat{\sigma}_{g\varepsilon}^{2}=1$). Parameter estimates can subsequently be scaled to approximate alternative $\sigma_{g\varepsilon}^{2}$ values, such as $\sigma_{g\varepsilon}^{2}=0$, for the Bernoulli mean-variance relationship (see below).

We inferred the grammar(s) subjects utilized by considering the relative likelihood of $p_{g}$ across the $k$ grammars hypothesized for each experimental session. Consider the random variable $Y_{rule}$ describing expected responses for the grammar rule employed by the average participant across trials in an experimental session, which we assume is equivalent to one of the hypothesized grammars. If participants correctly applied the rule corresponding to $Y_{rule}$, then $p_{rule}$ should be high and, consequentially, $p_{g}$ with larger values are more likely to parameterize $Y_{rule}$. We therefore expected $p_{rule}=\max{(p}_{g})$ and predicted that $p_{t}>p_{g-t}$ for target grammar $t$ and alternative grammars $g-t$. If $Y_{rule}\neq Y_{g}$ and the grammar described by $Y_{g}$ is not nested within $Y_{rule}$, then $Y_{g}$ should describe randomly consistent responses ($p_{g}=0.5$).

Within this framework, we pursued two primary forms of hypothesis testing. First, we considered whether $p_{g}=0.5$, predicting that target grammar $t$ would exhibit the most accurate performance ($p_{t}>p_{g-t}$ and $p_{t}>0.5$) while a large proportion of alternative grammars $g-t$ would be consistent with random responses ($p_{g-t}\approx0.5$). Secondly, we considered the relative odds of $t$ in comparison to$g-t$ for each experimental session. Log odds were used for all comparisons to correct for skewness in the odds ratio distribution. We expected that the relative odds of $t$ would be significantly greater than $g-t$, such that we predicted ${lOR}_{t,g-t}>0$ for the log odds ratio ($lOR$)

|  | ${lOR}_{t,g-t}={ln\left( \frac{\hat{p}_{t}}{1-\hat{p}_{t}} \right)}/{ln\left( \frac{\hat{p}_{g-t}}{1-\hat{p}_{g-t}} \right)}=\hat{\alpha}_{t}-\hat{\alpha}_{g-t}$ | (3) |
| --- | --- | --- |

Even in the case that ${lOR}_{t,g-t}>0$ and $p_{g-t}=0.5$, a subset of subjects may have employed alternative grammars rather than the target grammar. We therefore tested both average subject and subject specific hypotheses. Multilevel modeling can be used to simultaneously estimate both sets of parameters. This was done by estimating the degree of unobserved heterogeneity in (1) accounted for by among-subject variance across trials. We assumed the random deviance $U_{gj}$ of the expected response for subject $j (j=1,\ldots,m)$ from the average $\alpha_{g}$ was normally distributed with among-subject variance $\sigma_{gj}^{2}$ such that

$$U_{gj}{\sim N(0,\sigma}_{gj}^{2})$$

The trial response for subject $j$on grammar $g$ is therefore given by the GLMM

|  | $y_{gj}^{*}=\alpha_{g}+u_{gj}+\varepsilon_{gj}$ | (4) |
| --- | --- | --- |

with subject specific intercept $\alpha_{gj}$ given by

|  | $\alpha_{gj}=\alpha_{g}+u_{gj}$ | (5) |
| --- | --- | --- |

As the residual variance $\sigma_{g\varepsilon}^{2}$ cannot be estimated for $Y_{g}$ and is fixed to unit variance, approximations (Diggle, Heagerty, Liang, & Zeger, 2004) can be used for (4) to marginalize over $\sigma_{g\varepsilon}^{2}$

|  | $E_{\sigma_{g\varepsilon}^{2}}\left( Y_{g} \right)\approx{logit}^{-1}\left( \frac{\alpha_{g}}{1+c^{2}\sigma_{g\varepsilon}^{2}} \right)$ $c=16\sqrt{3}/(15\pi)$ | (7) |
| --- | --- | --- |

and scale model parameters based on the assumption that $\sigma_{g\varepsilon}^{2}=0$

|  | ${\alpha^{'}}_{gj}=\alpha_{gj}\sqrt{\frac{1+c^{2}\sigma_{g\varepsilon}^{2}}{1+c^{2}\hat{\sigma}_{g\varepsilon}^{2}}}={\alpha_{gj}}/{\sqrt{1+c^{2}\hat{\sigma}_{g\varepsilon}^{2}}}$ | (8) |
| --- | --- | --- |
|  | $\sigma_{gj}^{2'}=\sigma_{gj}^{2}\frac{1+c^{2}\sigma_{g\varepsilon}^{2}}{1+c^{2}\hat{\sigma}_{g\varepsilon}^{2}}=\sigma_{gj}^{2}/(1+c^{2}\hat{\sigma}_{g\varepsilon}^{2})$ | (9) |

Subject-level hypothesis testing for $lOR$s was carried out by substituting the logit-scale subject intercept $\hat{\alpha}_{gj}$ into (3). The random intercept model given by (4) provides more robust $\hat{\alpha}_{gj}$ than either subject-specific or fixed effects logistic regression models. This is due to the so-called partial pooling, or shrinkage, of subject parameters toward the average intercept, where the degree of pooling is inversely proportional to the reliability of repeated subject measurements (Gelman & Pardo, 2006). Through parameter shrinkage, mixed effects models appropriately reduce the inferential risks associated with multiple comparisons (Gelman, Hill, & Yajima, 2012) as well as traditional, overly conservative adjustment procedures such as Bonferroni correction (Nakagawa, 2004).

In addition to our primary hypotheses, we further considered among-subject variability in subjects’ performance for the target grammars across experimental sessions. If subjects exhibited individual differences in their ability to acquire and/or appropriately apply the target grammar $t$, then evidence of consistent among-individual variance ($\sigma_{tj}^{2}>0$) in performance should be detected across trials. Moreover, if among-subject variance in the target grammars reflects individual differences in some more generalized cognitive ability, covariance between individual performance across the target grammars should be detected. Whether this is a general motivational or attentional variable, or something more specific to pattern perception, cannot be determined given our data.

The intraclass correlation coefficient ($ICC$) provides a standardized metric of the proportion of observed variance explained by variability in subject specific intercepts. Following Nakagawa & Schielzeth (2010), we calculated ${ICC}_{t}$ as

|  | ${ICC}_{t}={\sigma_{tj}^{2}}/\left( \sigma_{tj}^{2}+\sigma_{t\zeta}^{2} \right)$  $\sigma_{t\zeta}^{2}=\sigma_{t\varepsilon}^{2}+\pi^{2}/3$  $\sigma_{t\zeta}^{2}=\pi^{2}/3$ | (10) |
| --- | --- | --- |

with the latent scale residual variance $\sigma_{t\zeta}^{2}$ determined by the logistic distribution variance $\pi^{2}/3$ due to the assumption that $\sigma_{t\varepsilon}^{2}=0$. Given $\sigma_{tj}^{2}>0$, we further specified a multivariate GLMM for the **Copy** ($t1$), **Mirror** ($t2$), and **AB^n^A** ($t3$) target grammars

|  | $y_{t1j}^{*}=\alpha_{t1}+u_{t1j}+\varepsilon_{t1j}$  $y_{t2j}^{*}=\alpha_{t2}+u_{t2j}+\varepsilon_{t2j}$  $y_{t3j}^{*}=\alpha_{t3}+u_{t3j}+\varepsilon_{t3j}$ | (11) |
| --- | --- | --- |

with an unstructured among-subject variance-covariance matrix

|  | $\left[ \begin{matrix} u_{t1j} \\ u_{t2j} \\ u_{t3j} \end{matrix} \right] \sim MVN\left( 0,\Omega_{subject} \right) :$ $\Omega_{subject}=\left[ \begin{matrix} \begin{matrix} \sigma_{t1j}^{2} \\ \sigma_{u_{t2j},u_{t1j}} \\ \sigma_{u_{t3j},u_{t1j}} \end{matrix} & \begin{matrix} \sigma_{u_{t1j},u_{t2j}} \\ \sigma_{t2j}^{2} \\ \sigma_{u_{t3j},u_{t2j}} \end{matrix} & \begin{matrix} \sigma_{u_{t1j},u_{t3j}} \\ \sigma_{u_{t2j},u_{t3j}} \\ \sigma_{t3j}^{2} \end{matrix} \end{matrix} \right]$ | (12) |
| --- | --- | --- |

Residual (co)variance was fixed to diagonal and parameters were subsequently adjusted using (8) and (9).

We utilized a Bayesian framework to estimate the parameters for (4) and (11) with Markov Chain Monte Carlo (MCMC) sampling. Bayesian MCMC sampling facilitates unbiased estimation of marginal parameter densities, overcoming the limitations of traditional frequentist approximations for the GLMM (Zhao, Staudenmayer, Coull, & Wand, 2006). Moreover, by directly quantifying parameter uncertainty, straightforward hypothesis testing can be conducted through direct manipulation of posterior samples and interpolation of their densities. This approach obviates the contentious calculation of degrees of freedom for GLMMs (Bolker et al., 2009) and considers the degree of uncertainty in subject random effects, which is a nontrivial issue in frequentist models (Hadfield, Wilson, Garant, Sheldon, & Kruuk, 2010). Below we utilize the 95% highest posterior density to calculate Bayesian credibility intervals, and we consider an effect to be statistically significant whenever the 95% CI excludes alternative hypotheses.

**Text S2.** Fitting the univariate GLMM

Below are the coded responses of Subject ‘1’ in the experimental session for target grammar **Copy** along with the four alternative grammars **AFirst**, **ALast**, **BFirst**, and **BLast** shown.

*#load dataset*

copy<-read.csv("copy.csv") *#copy session grammars indexed by '...c'*

head(copy)[,1:7]

subject seq Afirst.c ALast.c BFirst.c BLast.c Copy.c

1 1 ABAB 1 0 0 1 1

2 1 ABAB 1 0 0 1 1

3 1 ABAB 1 0 0 1 1

4 1 ABAB 1 0 0 1 1

5 1 ABAB 1 0 0 1 1

6 1 BABA 0 1 1 0 1

As previously noted, responses consistent with a grammar rule are coded as ‘1’ while inconsistent responses are coded as ‘0’. For this tutorial, we compare the expected probability and relative log odds for the target **Copy** grammar and 14 alternative grammar response vectors. We begin by loading the R packages utilized throughout the rest of the tutorial: ‘MCMCglmm’ (Hadfield, 2010), ‘HDInterval’ (Meredith & Kruschke, 2016), ‘reshape2’ (Wickham, 2007), and ‘ggplot2’ (Wickham, 2009). We utilize ‘pacman’ (Rinker & Kurkiewicz, 2017) to efficiently load and update these packages.

library**(**pacman**)**

p_load**(**MCMCglmm,HDInterval,reshape2,ggplot2**)**

Rather than individually programming grammar models and subsequent comparisons, we utilize a simple for() loop function to efficiently complete this process. A matrix is first created to hold estimates of the average subject probability of success and relative log odds comparing the target to alternative grammar models.

*#probability of consistent response ('prob'), log odds ratio ('lOR'),
#and 95% highest posterior density CIs (‘lCI’, ‘uCI’)*

columns<-c("prob","problCI","probuCI","logOR","ORlCI","ORuCI")

*#grammar names (excluding [,1:2]: 'subject','seq')*

copy.grammars<-c(colnames(copy)[3:17])

copy.grammars *#15 total*

*#"Afirst.c" "ALast.c" "BFirst.c" "BLast.c" "Copy.c" "CpyA.4.c" #"CpyA.6.c" "CpyA.4.c.1" "CpyA.6.c.1" "CpyB.4.c" "CpyB.6.c"*

*#"CpyB.4.c.1" "CpyB.6.c.1" "SomeA.c" "SomeB.c"*

*#create matrix to hold model parameters and 95% CIs*

copy.avg.gram<-matrix(NA,15,6,dimnames=list(copy.grammars,columns))

Fitting Bayesian GLMMs requires the specification of prior distributions for all model parameters. Recall that the univariate model parameters are described by (4) in **Text S1**. The prior for this model contains B (fixed effect), G (random effect), and R (residual/error) matrices. We use a diffuse normal prior where $\mu=0$ and $\sigma^{2}={10}^{8}$ for the average subject intercept, which is the default prior for fixed effects in ‘MCMCglmm’ and is therefore not specified below. So-called parameter expanded priors from the non-central scaled F-distribution are specified for the subject random effect variance, which enhance MCMC mixing properties in comparison to the traditional inverse Wishart prior (Gelman, 2006). As described above, residual variance cannot be estimated for (4). We therefore fix it to unit variance for estimation.

prior.bern <- list(G=list(G1=list(V=1,nu=1,alpha.mu=0,alpha.V=1000)),R=list(V=1,fix=1))

Note that the use of regularizing priors, which place less prior probability upon implausible values, can be used to enhance the conservatism and efficiency of model fitting (McElreath, 2016), particularly for the estimation of boundary cases such as probabilities near the limits of (0,1) for a binary response variable.

The basic structure of each model is

g.mod<-MCMCglmm(g ~ 1,random=~subject, prior=prior.bern,

family="categorical", data=copy, nit=520000, thin=1, burnin=20000, verbose=FALSE, pr=TRUE)

for grammar $g$ in the copy.grammars vector. All models are specified with the ‘categorical’ family, which corresponds to the logistic Bernoulli error structure appropriate for binary responses. The argument pr=TRUE ensures that the individual random effect estimates are saved. Posterior estimates were generated using 520,000 MCMC samples (nit), and 20,000 were discarded to facilitate stabilization of the initial chain (burnin). Note that thinning of the MCMC chain (thin > 1) is inefficient for summarizing posterior distributions and should generally be avoided unless extensive post-processing of MCMC samples is required (Link & Eaton, 2012). The MCMC sampling properties specified here have been pretested to ensure appropriate stability and converge in the MCMC chain, as well as to generate effective sample sizes of at least 1000 for each intercept parameter. We do not cover such model diagnostics here, as extensive resources are available online. Although the appropriate settings will vary across models, it is important to ensure that the size of MCMC chains is constant across models for appropriate posterior comparisons. Note that parameter estimation using Hamiltonian Monte Carlo (HMC) tends to be much more computationally efficient that the Gibbs sampler utilized for model fitting in ‘MCMCglmm’. The recently developed Stan (Stan Development Team, 2017) statistical environment implements HMC and can be interfaced through R using accessible packages such as ‘brms’ (Bürkner, 2016).

After estimating a model, we scale the posterior average subject intercept and random effects (contained in g.mod$Sol) as well as the random effect variance component (contained in g.mod$VCV) to approximate a joint distribution with zero residual variance. This is done using (8) and (9), which rely upon the squared constant $c$ defined in (7) as well as the estimated residual variance labelled units in the MCMCglmm output (g.mod$VCV[,2]).

c2 <- ((16 * sqrt(3))/(15 * pi))^2

g.Sol<- g.mod$Sol/sqrt(1 + c2 * g.mod$VCV[,2])

g.VCV<-g.mod$VCV/(1 + c2 * g.mod$VCV[,2])

We then convert the logit scale average subject intercept to the probability scale using (2) and summarize the mean and 95% highest posterior density (HPD) of this $p_{g}$ distribution.

g.odds<-exp(g.Sol[,1])

g.prob<-g.odds/(1+g.odds)

mean(g.prob)

hdi(g.prob)

We are now ready to create a loop to estimate the model parameters necessary for both average subject and subject specific hypothesis testing. We use the assign() function throughout to create permanent model objects using the relevant grammar name. Note that computing many lengthy chains as specified below will utilize a large quantity of RAM, and researchers may opt to modify our approach using various big data packages in R or simply compare models piecemeal depending upon their available hardware. As previously noted, HMC estimation also tends to be more computationally efficient than the Gibbs sampler utilized below.

*#computationally intensive (console may hang for hours)*

for(g in copy.grammars){

*#set pseudorandom number generator for replicability*

set.seed(9)

*#fixed effect formula for average subject intercept 'g ~ 1'*

fixed<-as.formula(substitute(g ~ 1,list(g=as.name(g))))

g.mod<-MCMCglmm(fixed,random=~subject,prior=prior.bern,slice=T,

family="categorical",data=copy, nit=520000, thin=1, burnin=20000, verbose=FALSE,pr=TRUE)

assign(paste0(g,"mod"),g.mod)

g.Sol<- g.mod$Sol/sqrt(1 + c2 * g.mod$VCV[,2])

assign(paste0(g,"Sol"),g.Sol)

g.VCV<-g.mod$VCV/(1 + c2 * g.mod$VCV[,2])

assign(paste0(g,"VCV"),g.VCV

g.odds<-exp(g.Sol[,1])

g.prob<-g.odds/(1+g.odds)

assign(paste0(g,"prob"),g.prob)

*#put results in matrix*

copy.avg.gram[g,1]<-mean(g.prob)

copy.avg.gram[g,2:3]<-hdi(g.prob)

}

After running this loop, we have estimates for all parameters needed to conduct both subject average and subject specific hypothesis testing. We first ensure that we have generated a sufficient effective sample size for the intercept parameters across our models. We create a matrix to hold our effective sample size estimates and then populate it using the effectiveSize() function.

ES.copy<-matrix(NA,15,21,dimnames=list(copy.grammars,c("Intercept",paste0("sub",1:20))))

for(g in copy.grammars){

ES<-effectiveSize(eval(parse(text=paste0(g,"mod$Sol"))))*#string to variable name*

ES.copy[g,]<-ES

}

*#Check for (<1000 independent samples)*

ES.copy**[**apply**(**ES.copy,MARGIN**=**1,function**(**x**)** any**(**x **<** 1000**))**,**]**

An empty matrix is returned, which suggests that we have sufficient effective sample sizes to begin model comparisons. We now turn to calculating the log odds ratios ($lORs$) comparing the target **Copy** grammar to the alternative set using (3). We summarize the log odds posterior using the mean and 95% HPD.

for(g in copy.grammars){

OR<-log(Copy.codds)-log(get(paste0(g,"odds")))

copy.avg.gram[g,4]<-mean(OR)

copy.avg.gram[g,5:6]<-hdi(OR)

}

We can now view the results of our hypothesis tests, with significant effects being indicated by 95% that do not overlap alternative hypotheses. Recall for the target grammar $t$ compared to the alternative grammars $g-t$, we predicted $p_{g-t}=0.5$, $p_{t}>p_{g-t}$, and ${lOR}_{t,g-t}>0$.

copy.avg.gram

prob problCI probuCI logOR ORlCI ORuCI

Afirst.c 0.4526602 0.4267708 0.4789871 1.985424 1.4081515 2.560005

ALast.c 0.4850351 0.4594158 0.5105448 1.855278 1.2788388 2.428374

BFirst.c 0.5473698 0.5210071 0.5731935 1.605174 1.0314071 2.182961

BLast.c 0.5149085 0.4894200 0.5405418 1.735667 1.1634824 2.313574

Copy.c 0.8540741 0.7837064 0.9204790 0.000000 0.0000000 0.000000

CpyA.4.c 0.5128961 0.4412640 0.5840226 1.743494 1.1172568 2.382513

CpyA.6.c 0.5081966 0.4359362 0.5814112 1.762391 1.1229809 2.392862

CpyA.4.c.1 0.5128961 0.4412640 0.5840226 1.743494 1.1172568 2.382513

CpyA.6.c.1 0.5081966 0.4359362 0.5814112 1.762391 1.1229809 2.392862

CpyB.4.c 0.5092378 0.4341855 0.5829086 1.758191 1.1204017 2.397196

CpyB.6.c 0.5156760 0.4443063 0.5864224 1.732310 1.1058842 2.369052

CpyB.4.c.1 0.5092378 0.4341855 0.5829086 1.758191 1.1204017 2.397196

CpyB.6.c.1 0.5156760 0.4443063 0.5864224 1.732310 1.1058842 2.369052

SomeA.c 0.5427389 0.4617132 0.6250416 1.622793 0.9708037 2.279584

SomeB.c 0.5427389 0.4617132 0.6250416 1.622793 0.9708037 2.279584

Our hypotheses are generally confirmed across all grammars, although the **BFirst** alternative grammar exhibits a slightly above chance response probability. It’s easier to see the $lOR$ results by plotting a simple, unformatted figure with the ‘ggplot2’ package.

*#less memory efficient data frame object needed for 'ggplot' package*

group.copy<-data.frame(copy.avg.gram)

*#exclude Copy/Copy*

group.copy<-group.copy[-(which(rownames(group.copy)=="Copy.c")),]

ggplot(group.copy, aes(x=rownames(group.copy), y=logOR, fill=rownames(group.copy))) +

geom_bar(stat="identity",alpha=0.7,color="black",position=position_dodge())+

geom_errorbar(aes(ymin=ORlCI, ymax=ORuCI),width=.2,size=1,position=position_dodge(0.9))+
xlab("Copy / Alternative Grammar")+

ylab("Log Odds Ratio")+

ggtitle("Average Copy Grammar Log Odds Ratios") +

theme(axis.title.y=element_text(size=13),axis.text.y=element_text(size=10),

axis.title.x=element_text(size=13),axis.text.x=element_blank(), axis.ticks.x=element_blank(),

lot.title=element_text(size=13,hjust = 0.5))+ guides(fill=guide_legend(title="Alternative Grammars"))

Finally, we calculate the intraclass correlation coefficient for the target **Copy** grammar using (10).

*#denominator=subject variance + 0 residual variance + logistic variance*

Copy.cICC<-Copy.cVCV[,1]/(Copy.cVCV[,1]+pi^2/3)

mean(Copy.cICC)

[1] 0.3013296

hdi(Copy.cICC)

var1

lower 0.1552414

upper 0.4627089

attr(,"credMass")

[1] 0.95

**Text S3**. Subject specific estimates

Having shown that the target **Copy** grammar is likely to have been employed by the average subject during our experimental session, we now examine which grammars are most consistent with the performance of individual subjects. We first create a matrix to hold the subject specific expected and 95% CI $lOR$ estimates.

ind.copycol<-c(rbind(paste0(copy.grammars,"logOR"),paste0(copy.grammars,"lCI"),paste0(copy.grammars,"uCI")))

*#20 subjects, 15 grammars x 3 values*

ind.copy<-matrix(NA,20,45,dimnames=list(1:20,ind.copycol))

We then run another loop to compare the relative odds of subject $j$’s **Copy** grammar and

alternative grammar performance.

for(g in copy.grammars) {

for(j in 1:20) {

gram<-(get(paste0(g,"Sol")))

lnOR<-(Copy.cSol[,1]+Copy.cSol[,eval(j+1)])-(gram[,1]+gram[,eval(j+1)])

assign(paste0(g,j,"lnOR"),lnOR)

ind.copy[j,paste0(g,"logOR")]<-mean(lnOR)

ind.copy[j,paste0(g,"lCI")]<-hdi(lnOR)[1]

ind.copy[j,paste0(g,"uCI")]<-hdi(lnOR)[2] }

}

To visualize these results, we first transform the matrix into a long format data frame appropriate for ‘ggplot2’ using the melt() function in ‘reshape2’.

OR.copy<-reshape2:::melt.array(ind.copy[,grepl("logOR",colnames(ind.copy))],id=rownames,varnames=c("ID","grammar"),value.name = "logOR")

lCI.copy<-reshape2:::melt.array(ind.copy[,grepl("lCI",colnames(ind.copy))],id=rownames,varnames=c("ID","grammar"),value.name = "lCI")

uCI.copy<-reshape2:::melt.array(ind.copy[,grepl("uCI",colnames(ind.copy))],id=rownames,varnames=c("ID","grammar"),value.name = "uCI")

*#combine dataframes*

long.copy<-cbind(OR.copy,lCI.copy,uCI.copy)

*#remove duplicate columns*

long.copy<-long.copy[,!duplicated(colnames(long.copy))]

*#add "subject" to remaining ID column*

long.copy[,1]<-factor(rep(paste("subject",1:20),15),levels=unique(paste("subject",1:20)))

*#add grammar names to grammar*

long.copy[,2]<-factor(rep(copy.grammars,each=20))

*#exclude Copy / Copy*

long.copy<-long.copy[!grepl("Copy.c",long.copy$grammar),]

The data are now structured for easy subject specific visualization. The facet_wrap(~ID) argument produces distinct plots for each subject.

ggplot(long.copy, aes(x=grammar, y=logOR, fill=grammar)) + geom_bar(stat="identity",alpha=0.7,color="black",position=position_dodge())+

geom_errorbar(aes(ymin=lCI, ymax=uCI),width=.2,size=1,position=position_dodge(0.9))+

xlab("Copy / Alternative Grammar")+

ylab("Log Odds Ratio")+

ggtitle("Subject Copy Grammar Log Odds Ratios") + theme(axis.title.y=element_text(size=13),axis.text.y=element_text(size=10),

axis.title.x=element_text(size=13),axis.text.x=element_blank(), axis.ticks.x=element_blank(),

plot.title=element_text(size=13,hjust = 0.5))+ facet_wrap(~ID)+

guides(fill=guide_legend(title="Alternative Grammars"))

**Text S4**. Fitting the multivariate GLMM

Having now completed univariate model comparisons, we turn to considering the possible relationship between subjects’ target grammar performance across experimental sessions. Moderate among-subject variance was detected for the **Copy**, **Mirror**, and **AB^n^A** grammars. Here we investigate whether among-individual variance across experimental sessions reflects individual differences in a more generalized cognitive ability, which would be indicated by positive covariance between individual performance across the target grammars. We begin by loading and combining the grammar datasets.

*#load datasets*

copy<-read.csv("copy.csv")

mirror<-read.csv("mirror.csv")

abna<-read.csv("abna.csv")

*#join datasets into single data frame*

dataset<-cbind(copy,mirror,abna)
colnames(dataset)[1]<-"ID" *#change name for subject index column to 'ID'*

*#remove duplicate columns*

duplicates<-c("subject","seq")

dataset <- dataset[, !(names(dataset) %in% duplicates)]

We specify a multivariate prior using the same distributions discussed in **Text S2**.

prior.bern.mult <- list(G=list(G1=list(V=diag(3),nu=3,alpha.mu=rep(0,3),alpha.V=diag(3)*1000)),
R=list(V=diag(3),fix=1))

The multivariate model given by (11) and (12) can now be estimated. The us(trait):ID argument specifies an unstructured covariance matrix for subject random effects, and the idh(trait):units argument in combination with the fixed unit variance prior forces a diagonal residual variance. The MCMC chain settings were selected to ensure >1000 effective sample size for all parameters in (12). We do not save random effect estimates as our hypotheses concern the variance components.

set.seed(9)

multigram.mod<- MCMCglmm(cbind(Copy.c,Mirror.m,ABnA.a)~trait-1,

random=~us(trait):ID, rcov=~idh(trait):units,prior=prior.bern.mult,

family=rep("categorical",3),

data=dataset, nit=1020000, thin=1, burnin=20000, verbose=FALSE,pr=FALSE)

Posterior variance components are transformed using (9).

*#$VCV[,10:14] equivalent residual variance components fixed to 1*
multigram.VCV<-multigram.mod$VCV/(1 + c2 * multigram.mod$VCV[, 10])

We then create a data frame containing the posterior estimates of (12), which are standardized as Pearson correlation coefficients using the posterior.cor()function in ‘MCMCglmm’ to enhance interpretability. The mean and 95% HPD of these correlations is summarized to assess whether among-subject performance across target grammars is positively correlated.

*#[,1:9] are components in eq. (12)*
multi.cor**<-**data.frame**(**posterior.cor**(**multigram.VCV[,1:9]))

colnames**(**multi.cor**)<-**colnames**(**multigram.mod$VCV[,1:9])

colMeans(multi.cor)

traitCopy.c.1:traitCopy.c.1.ID traitMirror.m.1:traitCopy.c.1.ID

1.0000000 0.4684352

traitABnA.a.1:traitCopy.c.1.ID traitCopy.c.1:traitMirror.m.1.ID

0.5918419 0.4684352

traitMirror.m.1:traitMirror.m.1.ID traitABnA.a.1:traitMirror.m.1.ID

1.0000000 0.5230203

traitCopy.c.1:traitABnA.a.1.ID traitMirror.m.1:traitABnA.a.1.ID

0.5918419 0.5230203

traitABnA.a.1:traitABnA.a.1.ID

1.0000000

hdi(multi.cor)

traitCopy.c.1:traitCopy.c.1.ID traitMirror.m.1:traitCopy.c.1.ID

lower 1 0.08325313

upper 1 0.81243344

traitABnA.a.1:traitCopy.c.1.ID traitCopy.c.1:traitMirror.m.1.ID

lower 0.2486446 0.08325313

upper 0.8852669 0.81243344

traitMirror.m.1:traitMirror.m.1.ID

lower 1

upper 1

traitABnA.a.1:traitMirror.m.1.ID traitCopy.c.1:traitABnA.a.1.ID

lower 0.1457853 0.2486446

upper 0.8542910 0.8852669

traitMirror.m.1:traitABnA.a.1.ID traitABnA.a.1:traitABnA.a.1.ID

lower 0.1457853 1

upper 0.8542910 1

attr(,"credMass")

[1] 0.95

These results suggest a more generalized cognitive ability may cause individual differences in subject performance across experimental sessions. Whether this is a general motivational or attentional variable, or something more specific to pattern perception, cannot be determined given our data.

References

Bolker, B. M., Brooks, M. E., Clark, C. J., Geange, S. W., Poulsen, J. R., Stevens, M. H. H., & White, J. S. S. (2009). Generalized linear mixed models: A practical guide for ecology and evolution. *Trends in Ecology & Evolution*, *24*, 127-135.

Bürkner, P. C. (2016). brms: An R package for Bayesian multilevel models using Stan. *Journal of Statistical Software*, *80*, 1-28.

Diggle, P, Heagerty, P. J., Liang, K., & Zeger, S. L. (2002). *Analysis of longitudinal data* (2nd edition). New York, NY: Oxford University Press.

Gelman, A. (2006). Prior distributions for variance parameters in hierarchical models. *Bayesian Analysis*, *1*, 515-534.

Gelman, A., Hill, J., & Yajima, M. (2012). Why we (usually) don't have to worry about multiple comparisons. *Journal of Research on Educational Effectiveness*, *5*, 189-211.

Gelman, A., & Pardoe, I. (2006). Bayesian measures of explained variance and pooling in multilevel (hierarchical) models. *Technometrics*, *48*, 241-251.

Hadfield, J. D. (2010). MCMC methods for multi-response generalized linear mixed models: the MCMCglmm R package. *Journal of Statistical Software*, *33*, 1-22.

Hadfield, J. D., Wilson, A. J., Garant, D., Sheldon, B. C., & Kruuk, L. E. (2010). The misuse of BLUP in ecology and evolution. *The American Naturalist*, *175*, 116-125.

Link, W. A., & Eaton, M. J. (2012). On thinning of chains in MCMC. *Methods in Ecology and Evolution*, *3*, 112-115.

Meredith, M., & Kruschke, J. (2016). HDInterval: Highest (Posterior) Density Intervals. R package version 0.1.3. <https://CRAN.R-project.org/package=HDInterval>

McElreath, R. (2016). *Statistical rethinking: A Bayesian course with examples in R and Stan*. Boca Raton, FL: Chapman & Hall/CRC.

Nakagawa, S. (2004). A farewell to Bonferroni: the problems of low statistical power and publication bias. *Behavioral Ecology*, *15*, 1044-1045.

Nakagawa, S., & Schielzeth, H. (2010). Repeatability for Gaussian and non‐Gaussian data: A practical guide for biologists. *Biological Reviews*, *85*, 935-956.

Rinker, T. W. & Kurkiewicz, D. (2017). pacman: Package Management for R. version 0.4.6. http://github.com/trinker/pacman

Skrondal, A., & Rabe-Hesketh, S. (2004). *Generalized latent variable modeling: Multilevel, longitudinal, and structural equation models*. Boca Raton, FL: CRC Press.

Skrondal, A., & Rabe-Hesketh, S. (2007). Redundant overdispersion parameters in multilevel models for categorical responses. *Journal of Educational and Behavioral Statistics*, *32*, 419-430.

Stan Development Team. (2017). *Stan Modeling Language Users Guide and Reference Manual*, Version 2.17.0.   http://mc-stan.org

Wickham, H. (2007). Reshaping Data with the reshape Package. *Journal of Statistical Software*, *21*, 1-20.

Wickham, H. (2009). *ggplot2: Elegant Graphics for Data Analysis*. New York, NY: Springer-Verlag New York.

Zhao, Y., Staudenmayer, J., Coull, B. A., & Wand, M. P. (2006). General design Bayesian generalized linear mixed models. *Statistical Science*, 21, 35-51.
