# Supplementary material for: Artificial Grammar Learning Capabilities in an Abstract Visual Task Match Requirements for Linguistic Syntax
Source: Front Psychol. 2018 Jul 24;9:1210. doi: 10.3389/fpsyg.2018.01210 (PMC6066649; doi:10.3389/fpsyg.2018.01210)
Supplement: Supplementary file 4 [file Table_4.DOCX]

**Figure S4.1**. Log Odds Ratios of Target and Alternate Grammars for the Average Subject


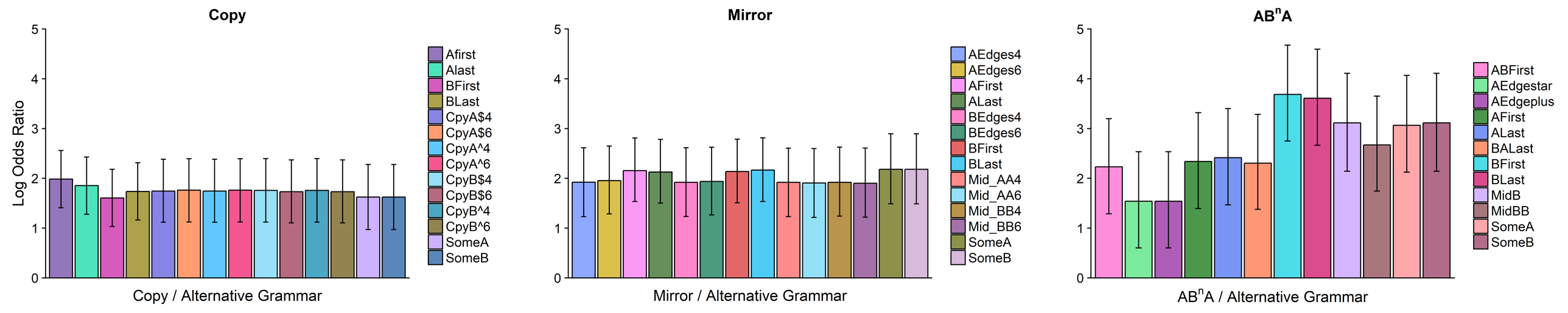


**Figure S4.2.** Log Odds Ratios of Copy and Alternate Grammars for Individual Subjects





**Figure S4.3.** Log Odds Ratios of Mirror and Alternate Grammars for Individual Subjects





**Figure S4.4.** Log Odds Ratios of ABNA and Alternate Grammars for Individual Subjects
